# *RAI1* safeguards fidelity and tempo of human neurodevelopmental gene expression

**DOI:** 10.64898/2026.06.11.731717

**Published:** 2026-06-12

**Authors:** Bo Zhou, Satabdi Mohanty, Paris Riggle, Takao Tsukahara, Grace Lin, Louis T. Dang, Michael A. Sutton, Shigeki Iwase

## Abstract

Human brain development proceeds on an unusually long timeline relative to other species, a feature that is thought to foster advanced cognitive abilities. Retinoic Acid Induced 1 ( *RAI1* ) gene encodes a nucleosome-binding protein haploinsufficient in Smith–Magenis Syndrome (SMS), a neurodevelopmental disorder characterized by cognitive impairment with autistic features. However, the role of *RAI1* in human neurodevelopment remains unexplored experimentally. Here, we generated isogenic heterozygous and homozygous *RAI1* loss-of-function human embryonic stem cell lines and interrogated the roles of *RAI1* in neurodevelopmental gene regulation. A longitudinal transcriptome analysis during *in vitro* cortical development revealed that *RAI1* deficiency accelerates developmental gene expression progression, including the precocious induction of synaptic genes. Single-cell RNA-seq analysis revealed that *RAI1-* deficient neuroprogenitors acquire a transient mesoderm-like gene expression signature followed by pro-neuronal maturation gene expression in postmitotic neurons. Unexpectedly, the developmental acceleration signature was exacerbated during NGN2-induced excitatory neuron differentiation, suggesting functional interplay between *RAI1* and NGN2-driven programs. Together, these results identify *RAI1* as a suppressor of the mesodermal lineage program and as a novel brake that slows the tempo of human neurodevelopmental gene expression.

## Full Text Availability

The license terms selected by the author(s) for this preprint version do not permit archiving in PMC. The full text is available from the preprint server.

