# Supplementary material for: RAI1 safeguards fidelity and tempo of human neurodevelopmental gene expression
Source: bioRxiv. 2026 Jul 28:2026.06.11.731717. Originally published 2026 Jun 12. Preprint. [Version 2] doi: 10.64898/2026.06.11.731717 (PMC13278167; doi:10.64898/2026.06.11.731717)
Supplement: Supplementary file 1 [file NIHPP2026.06.11.731717v2-supplement-1.pdf]

bioRxiv preprint doi: https://doi.org/10.64898/2026.06.11.731717; this version posted July 28, 2026. The copyright holder for this preprint (which was not certified by peer review) is the author/funder, who has granted bioRxiv a license to display the preprint in perpetuity. It is made available under a CC-BY 4.0 International license.

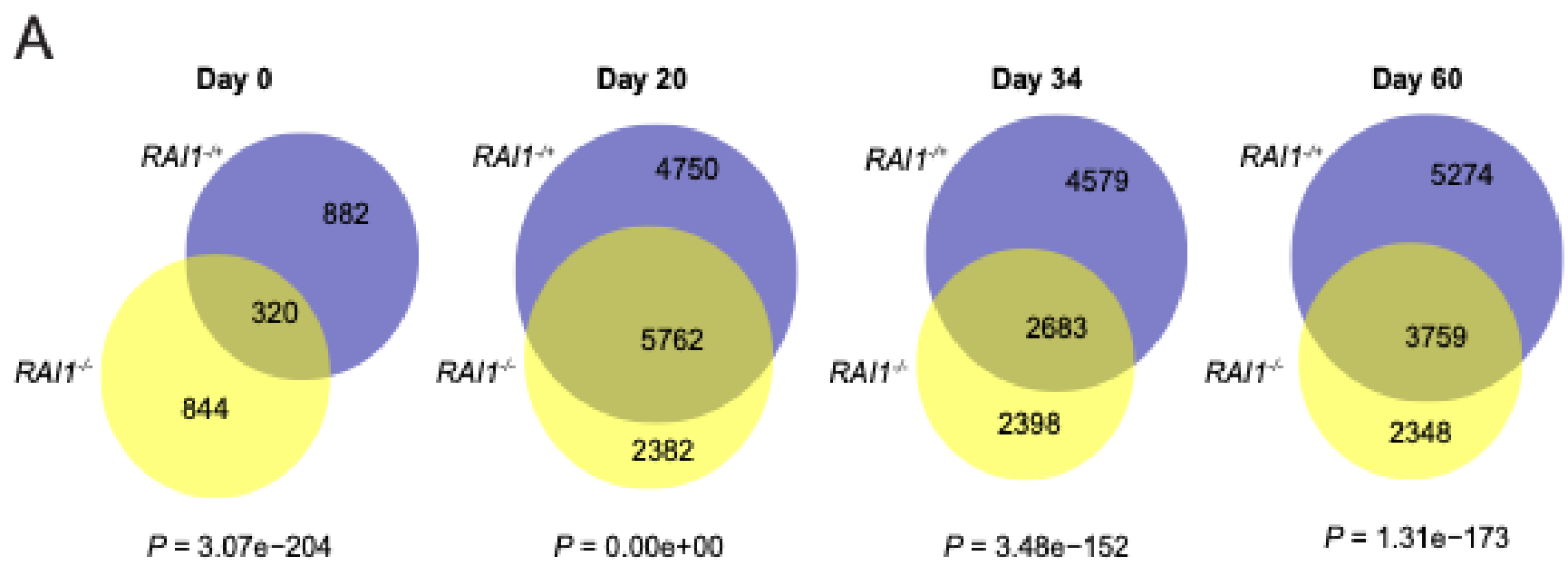

839

840 **Figure S2 : Differentially expressed genes overlap between *RAI1*+/- and *RAI1*-/- cells.**

841 **(A)** The number of DEGs (Padj < 0.05) in *RAI1*+/- and *RAI1*-/- compared to WT controls was denoted, and

842 their identities were compared. P-values of the chi-square test are denoted at the bottom of the Venn

843 diagrams.

844

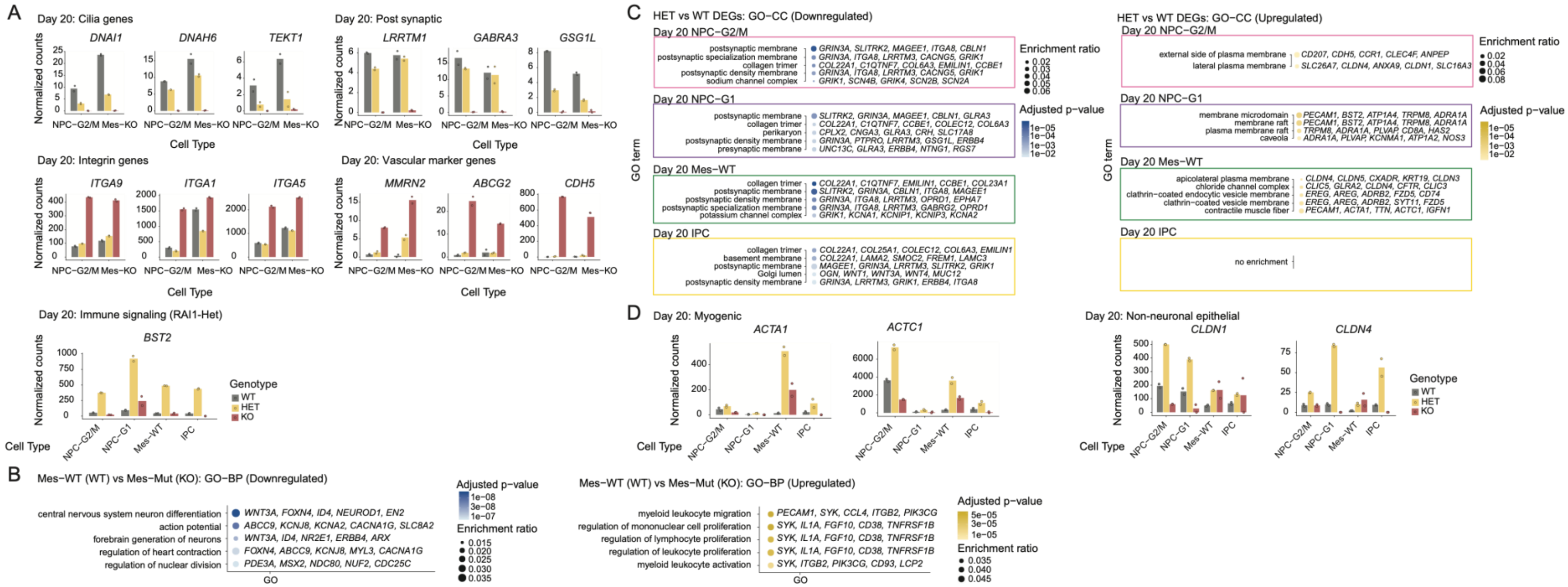

Figure S5: Additional analyses of genes dysregulated in *RAI1*-deficient cell types on day 20. (A) Expression of additional top contributors for GO term enrichment. **(B)** GO Cellular Component enrichment for *RAI1*-heterozygous DEGs. The top five GO terms are presented with the genes that contributed most to the enrichment. **(C)** GO Biological Processes enrichment analysis comparing Mes-WT (WT) and Mes-KO (KO). **(D)** Expression of the additional myogenic and non-neuronal epithelial genes across genotypes and cell types. Mean values are shown in histograms, with individual data points from biological duplicates.

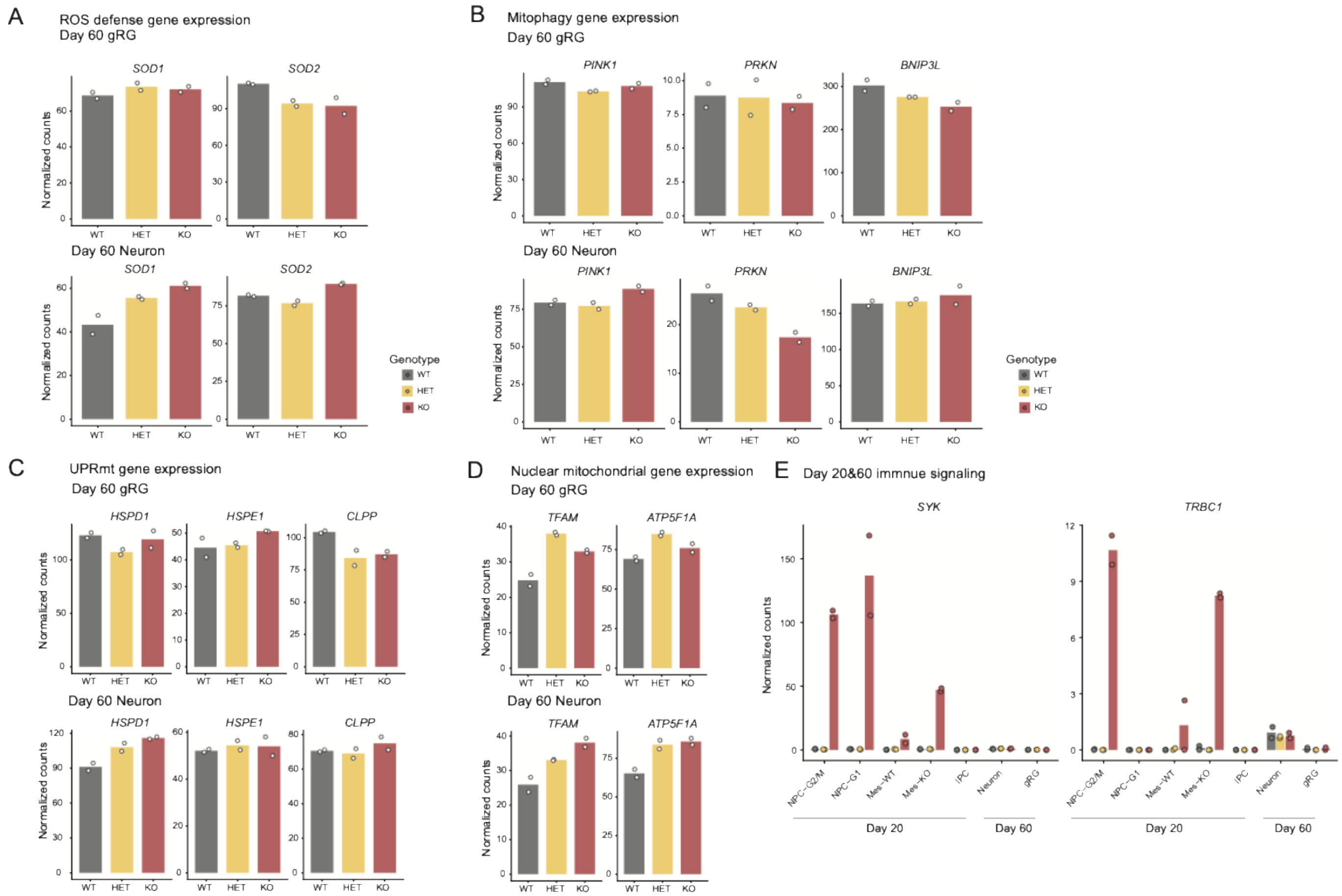

Figure S6: Additional gene expression plots of *RAI1*-deficient cell types on day 60.
(A) Expression of ROS scavengers. **(B)** Expression of mitophagy-related genes. **(C)** Expression of genes related to unfolded protein responses at mitochondria (UPRmt). **(D)** Expression of nuclear genome-encoded respiratory change regulator genes. Mean values are shown in histograms, with individual data points from biological duplicates. **(E)** Additional immune signaling genes were derepressed only in day 20 cells.

bioRxiv preprint doi: https://doi.org/10.64898/2026.06.11.731717; this version posted July 28, 2026. The copyright holder for this preprint (which was not certified by peer review) is the author/funder, who has granted bioRxiv a license to display the preprint in perpetuity. It is made available under a CC-BY 4.0 International license.

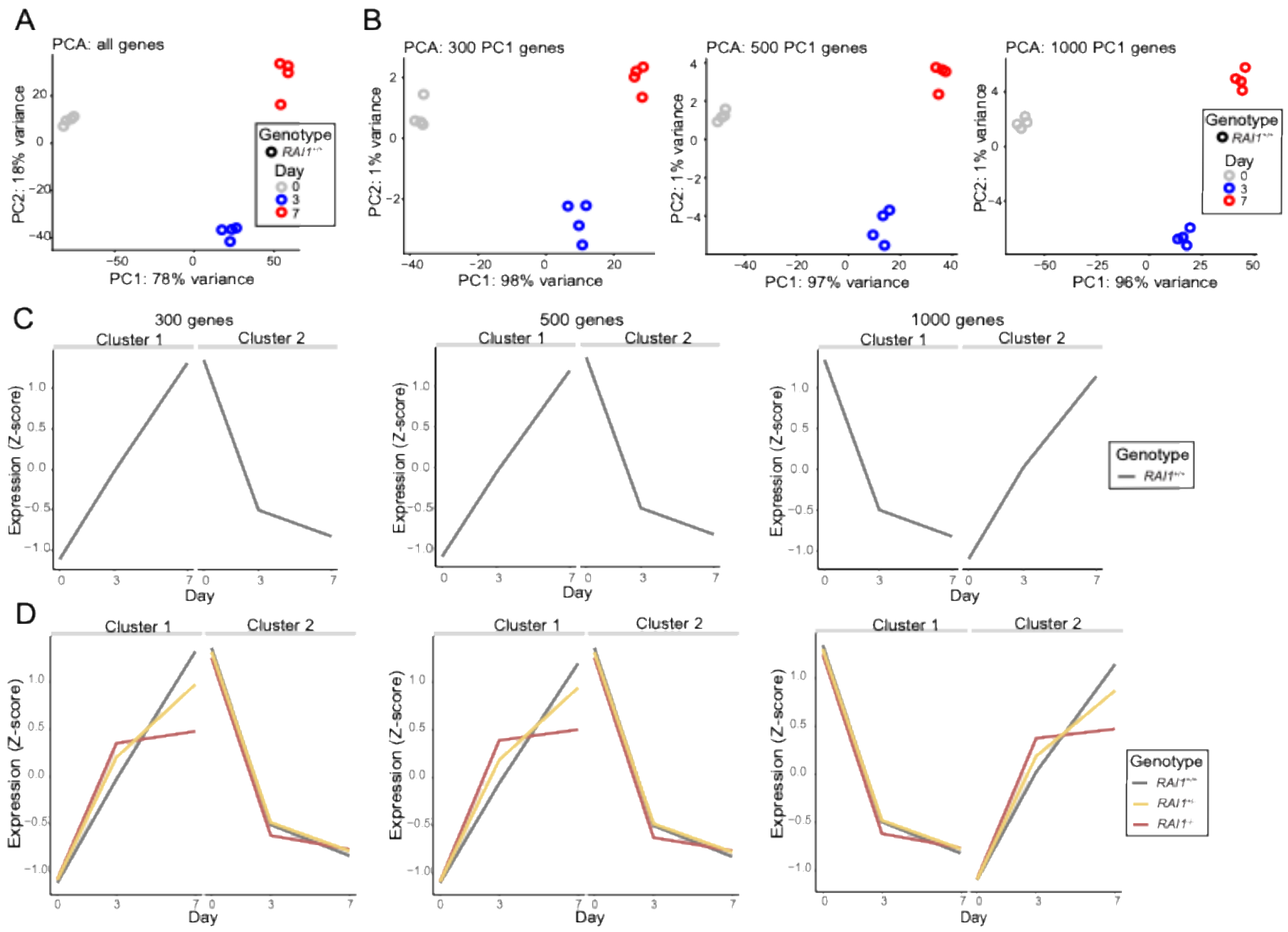

Figure S7: Principal component identification of core *Rai1*-dependent accelerated genes in iNeurons. **(A)** PCA of all genes in WT iNeurons. **(B)** PCA using the top 300, 500, and 1000 genes with the largest absolute gene loadings on PC1 from the plot in part A. **(C)** K-means clustering of gene expression in the top 300, 500, and 1000 genes with the greatest absolute value of gene loading on PC1 identified in part A. **(D)** Gene expression of WT, Het, and KO iNeurons within each cluster, identified from the corresponding number of top PC1 gene loadings shown in part B.

bioRxiv preprint doi: https://doi.org/10.64898/2026.06.11.731717; this version posted July 28, 2026. The copyright holder for this preprint (which was not certified by peer review) is the author/funder, who has granted bioRxiv a license to display the preprint in perpetuity. It is made available under a CC-BY 4.0 International license.

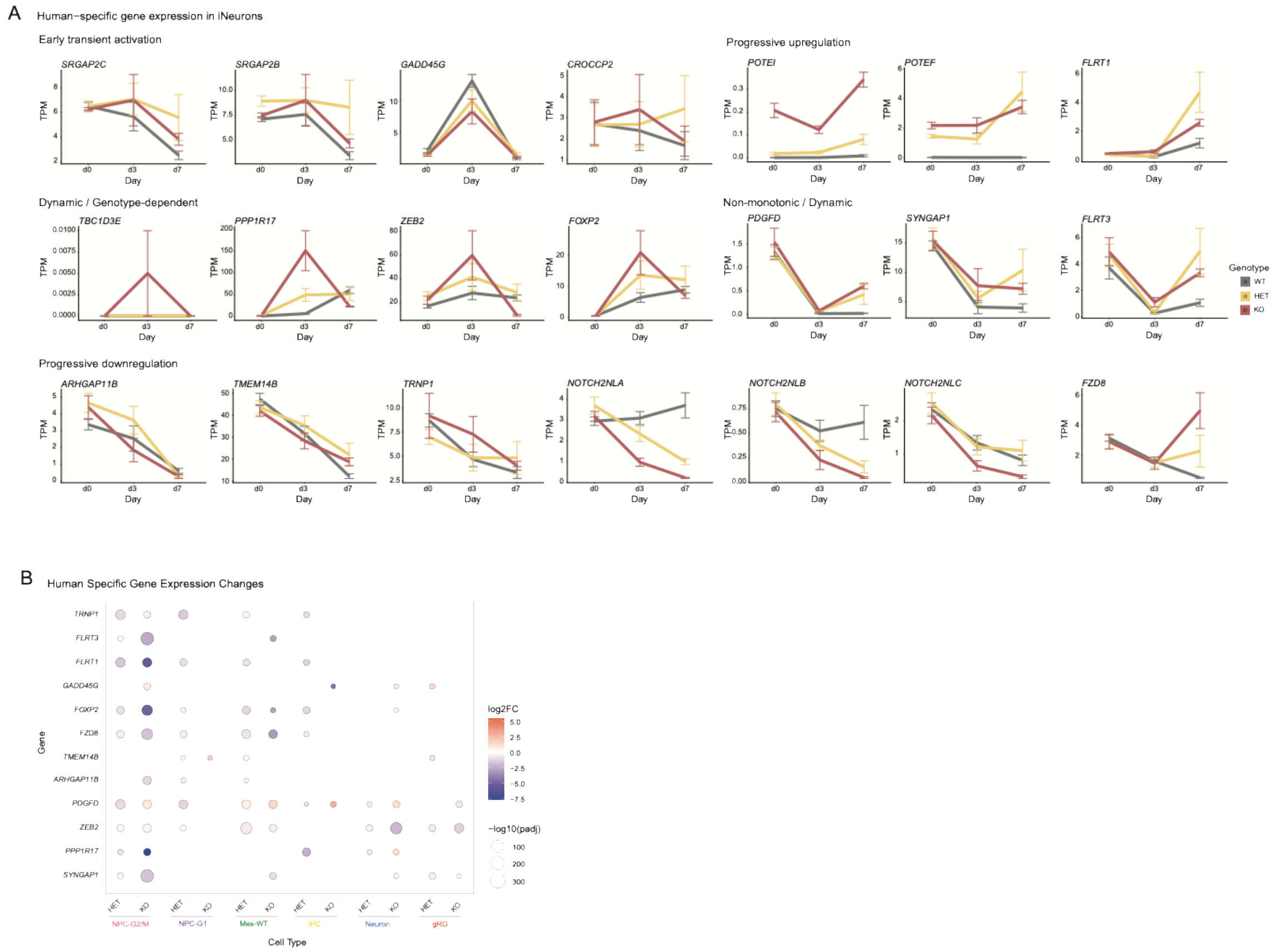

Fig S8: Expression of primate/human-specific genes implicated in the slower tempo of brain development.

(A) The genes are categorized based on how their expression changes in WT human iNeurons. **(B)** Expression of human primate-specific genes in single-cell RNAseq data. These genes are covered by the 10x Chromium Fixed RNA Profiling (Flex) genomic probes.
